# PHF6 maintains acute myeloid leukemia via regulating NF-κB signaling pathway

**DOI:** 10.1038/s41375-023-01953-6

**Published:** 2023-07-01

**Authors:** Shuaibing Hou, Xiaomin Wang, Tengxiao Guo, Yanjie Lan, Shengnan Yuan, Shuang Yang, Fei Zhao, Aizhong Fang, Na Liu, Wanzhu Yang, Yajing Chu, Erlie Jiang, Tao Cheng, Xiaojian Sun, Weiping Yuan

**Affiliations:** 1 State Key Laboratory of Experimental Hematology, National Clinical Research Center for Blood Diseases, Haihe Laboratory of Cell Ecosystem, Institute of Hematology & Blood Diseases Hospital, Chinese Academy of Medical Sciences & Peking Union Medical College, Tianjin, 300020 China; 2 Tianjin Institutes of Health Science, Tianjin, 301600 China; 3 Key laboratory of Carcinogenesis and Translational Research (Ministry of Education), Department of lymphoma, Peking University Cancer Hospital & Institute, Beijing, 100039 China; 4 Cancer Center, Beijing Tiantan Hospital, Capital Medical University, Beijing, 100070 China; 5 State Key Laboratory of Medical Genomics, Shanghai Institute of Hematology, National Research Center for Translational Medicine, Ruijin Hospital, Shanghai Jiao Tong University School of Medicine, Shanghai, 200025 China

**Keywords:** Acute myeloid leukaemia, Cell signalling

## Abstract

Acute myeloid leukemia (AML) is a major hematopoietic malignancy characterized by the accumulation of immature and abnormally differentiated myeloid cells in bone marrow. Here with in vivo and in vitro models, we demonstrate that the Plant homeodomain finger gene 6 (PHF6) plays an important role in apoptosis and proliferation in myeloid leukemia. Phf6 deficiency could delay the progression of RUNX1-ETO9a and MLL-AF9-induced AML in mice. PHF6 depletion inhibited the NF-κB signaling pathways by disrupting the PHF6-p50 complex and partially inhibiting the nuclear translocation of p50 to suppress the expression of BCL2. Treating PHF6 over-expressed myeloid leukemia cells with NF-κB inhibitor (BAY11-7082) significantly increased their apoptosis and decreased their proliferation. Taken together, in contrast to PHF6 as a tumor suppressor in T-ALL as reported, we found that PHF6 also plays a pro-oncogenic role in myeloid leukemia, and thus potentially to be a therapeutic target for treating myeloid leukemia patients.

## Introduction

Acute myeloid leukemia (AML) is a major hematopoietic malignancy characterized by the uncontrolled expansion of the immature myeloid cells [1]. The 5-year survival rates of young patients are 40–50%, while 5-year OS rates are less than 15% for patients aged over 60 [2, 3]. Much of the effort have been dedicated to decipher the molecular events underlying AML transformation, with the goals to identify specific therapeutic targets and develop new and more effective drugs. About half of AML patients had non-random chromosomal translocations, including balanced translocations, chromosomal gains or losses [4–6]. The four most common translocations are 11q23/mixed lineage leukemia (MLL)-fusion proteins, t(15;17)/PML-RAR, Inv(16)/core binding factor (CBF)b-MYH11 and t(8;21)/RUNX1-ETO [7]. These genetic changes lead to irreversible functional defects of critical genes that are associated with leukemogenesis. Clinical genetic evidences and experimental data from mouse models of leukemogenesis showed that more than one cooperating event were required to develop AML [8, 9].

Epigenetic dysregulation is a recurrent event in leukemogenesis [10]. When the expression of epigenetic regulators is altered, it would stimulate oncogenic transcriptional programming that are important for AML progression. Numerous evidences suggested that various AMLs are sensitive to the inhibition of these epigenetic regulators. For example, inhibition of TALE family homeodomain factor MEIS2 delayed the growth of RUNX1-ETO-positive leukemia cells [11]. Inactivation of the histone 3 lysine 79 methyltransferase Dot1l resulted in downregulation of MLL translocation-associated genes expression [12, 13]. Furthermore, inhibition of the arginine methyltransferase PRMT1 reduced the leukemic potential of several oncogenic fusion proteins, such as MLL-EEN and MLL-GAS7 [14, 15]. Notably, these epigenetic factors coordinated the regulation of gene expression in normal myelopoiesis while contributed to AML initiation and progression, thus providing a potential of therapeutic selectivity.

Plant homeodomain finger gene 6 (PHF6), is a highly conserved epigenetic transcriptional regulator that plays critical roles in neurodevelopment and hematopoiesis. It was first discovered to be mutated in patients with Börjeson–Forssman–Lehmann syndrome (BFLS) [16]. Additionally, BFLS patients had been reported to develop T-ALL disease [17]. Later, somatic mutations of PHF6 were reported in 38% of adult primary T-ALL cases [18] and 16–55% of mixed phenotype acute leukemia [19, 20]. Experimental data from mouse models showed that PHF6 plays a suppressive role in T-ALL leukemogenesis [21–23]. Interestingly, PHF6 mutations occur to a lesser extent, in 3% of acute myeloid leukemia, and 3% of high-grade B-cell lymphoma [24–26]. While Mousa et al. and de Rooij JD et al. studies showed that the expression of PHF6 was upregulated in AML patients, indicating it might be involved in the progression of AMLs [27, 28], the exactly functional role(s) of PHF6 in human myeloid leukemia remain unknown.

In the current study, we found that PHF6 is essential for the maintenance of self-renewal ability of leukemia stem cells (LSCs) but dispensable for hematopoietic stem cells (HSCs) in vivo. The survival of leukemia cells was sensitive to PHF6 depletion in both RUNX1-ETO9a (RE9a) and MLL-AF9 (MA9) AML mouse models and in human AML cell lines. Our data supports that both AML containing RUNX1-ETO9a and MLL-AF9 fusion proteins are dependent on PHF6 for their function, and that PHF6 may be a potential LSC-directed therapeutic target for AML.

## Methods

### Generation of AML mice and transplantation

*Phf6* conditional deletion mice were performed as described previously [22]. For RE9a-driven mouse model, the RE9a-GFP retroviral vector was kindly provided by Professor Xiaojian Sun. We used E14.5 fetal liver cells and performed the methods of Na Man et al. [29]. For MA9-driven mouse model, bone marrow of lineage negative (Lin^−^) cells from *Vav1-Cre;Phf6*^*fl/y*^ or *Phf6*^*fl/y*^ male mice were infected of MA9-GFP retroviral virus and constructed AML mice by the methods of Gao et al. [30]. All male mice weighed 20–30 g and aged 6–8 weeks. Animals were housed in the specific pathogen-free animal facility of the State Key Laboratory of Experimental Hematology (SKLEH), Institute of Hematology and Blood Disease Hospital. All experimental procedures for mice were approved by Laboratory Animal license for use (SYXK2020-0003). All efforts were made to minimize the suffering of the mice.

### Nuclear translocation assay

PHF6 KD Kasumi-1 and K562 cells with pre-treatment of starvation medium were centrifuged onto slides of treatment with Cell-TAK (Corning, New York, USA). 100 ng/mL TNF-α (Peprotech, New Jersey, USA) was added to the cells for 2 h. The cells were washed with PBS and fixed in 4% formaldehyde for 15 min. The cell membrane was permeabilized by 0.1% Triton in PBS for 15 min, and blocked by 2% BSA in PBS for 45 min. The cells were treated with anti-p65 or anti-p50 monoclonal antibody (dilution, 1:200, CST, Boston, MA, USA) for overnight at 4 °C. After washing with 0.5% BSA in PBS three times, the cells were stained with Alexa Fluor 647/594/488 goat anti-rabbit IgG (All dilution, 1:500, Invitrogen, CA, USA). The slides were then incubated with DAPI (BioLegend, CA,USA) for 5 min and observed on microscope (UltraVIEW VOX, Perkinelmer, Massachusetts, USA).

### NF-κB inhibitor experiments

For in vitro experiment, 7 × 10^4^/ml Kasumi-1 and K562 cells were treated with 4 μM Bay11-7082 (MCE, New Jersey, USA) or 2‰ DMSO in culture medium and then examined the apoptosis and proliferation of leukemia cells. For the in vivo experiment, 5 × 10^6^ PHF6 OE Kasumi-1 or control cells were transplanted into NSG mice irradiated with 200 cGy through tail vein injection. After a month, two groups of mice received 10 mg/kg Bay11-7082 by intraperitoneal injection of three times in 10 days. The NSG mice were purchased from Beijing HFK Bio-Technology and were aged 4–6 weeks.

## Results

### PHF6 is required for the growth of AML cells but dispensable for normal hematopoiesis

**Fig. 1:**
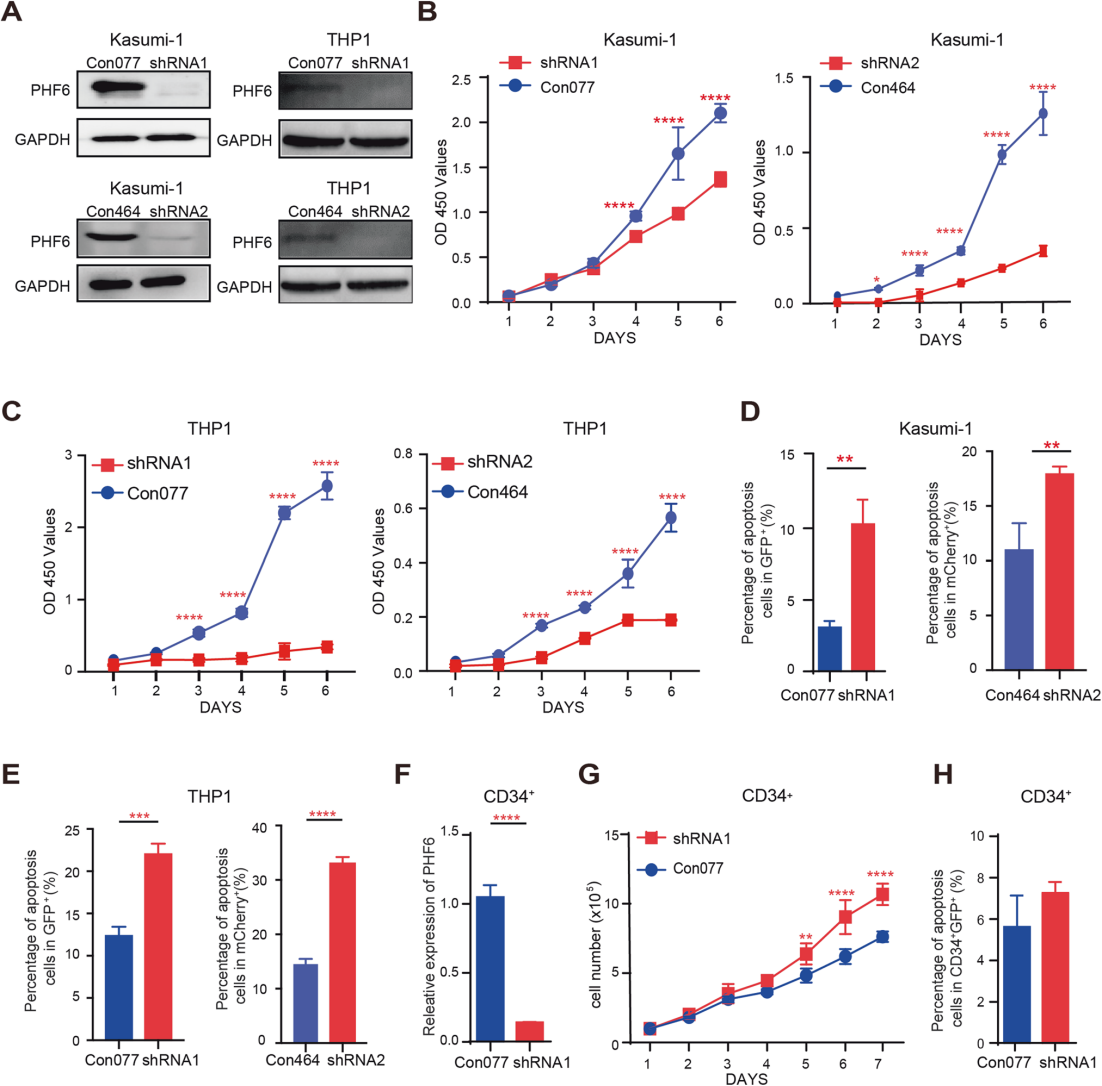
PHF6 KD inhibited the proliferation of myeloid leukemia cells, but had little effect on normal blood cells. **A** Knocking down PHF6 (PHF6 KD) in Kasumi-1 and THP1 cells by two different anti-PHF6 shRNAs. **B**, **C** The proliferation of PHF6 KD Kasumi-1 cells and PHF6 KD THP1 cells. **D**, **E** The percentage of apoptotic PHF6 KD Kasumi-1 and THP1 cells. **F** Knocking down PHF6 in cord blood CD34^+^ cells. **G** The proliferation of PHF6 KD (shRNA1) CD34^+^ cord blood cells. **H** The percentage of apoptotic CD34^+^ cord blood cells. Data are represented as mean ± SD.

To investigate the potential role of PHF6 in AML, we analyzed the relationship of PHF6 expression and overall survival. AML patients with high PHF6 expression had unfavorable prognosis than AML patients with low PHF6 expression (*p* = 0.0329) according to the survival time of patients with more than one year from the Cancer Genome Atlas AML dataset (Supplementary Fig. 1A). However, we did not detect obvious difference between bone marrow cells of AML patients and normal person (Supplementary Fig. 1B, C). To further confirm whether PHF6 was truly functional relevant to myeloid leukemia development, we firstly over-expressed PHF6 in Kasumi-1 and K562 cells (Supplementary Fig. 1D). We found that over-expression of PHF6 (PHF6 OE) increased the growth of Kasumi-1 and K562 cells (Supplementary Fig. 1E), decreased the apoptosis of Kasumi-1 cells while has no effect on K562 cells (Supplementary Fig. 1F). Furthermore, we knocked down PHF6 (PHF6 KD) in myeloid cell lines by two independent anti-PHF6 shRNA (Fig. 1A and Supplementary Fig. 1G). PHF6 KD significantly decreased the growth of Kasumi-1, THP1 and K562 cells (Fig. 1B, C and Supplementary Fig. 1H), and promoted cell apoptosis (Fig. 1D, E and Supplementary Fig. 1I). In addition, we performed the CFC assay to determine the impact on clonogenicity. PHF6 KD also decreased the colony number of Kasumi-1 and THP1 cells (Supplementary Fig. 1J, K). These results indicated that PHF6 might involve in myeloid leukemia initiation and progression.

Furthermore, we knocked down PHF6 in CD34^+^ cord blood cells and investigated the role of PHF6 in hematopoiesis (Fig. 1F). We found PHF6 KD increased the CD34^+^ cord blood cell proliferation and had little effects on cell apoptosis (Fig. 1G, H). To analyze the clonogenicity of PHF6 KD CD34^+^, we performed the CFC assays and found that the total colony number of PHF6 KD cells were increased than control groups. The major types of increased CD34^+^ cell derived colonies were CFU-E and BFU-E. It indicated that PHF6 KD promoted CD34^+^ cells proliferation and differentiation to erythroid cells in vitro (Supplementary Fig. 2A). Further, we generated *Vav1-Cre;Phf6*^*fl/y*^ (*VC Phf6*, PHF6 knock-out in blood cells) and *Phf6*^*fl/y*^ (*Phf6 WT)* mice and found that *Phf6* deletion had no influence on the number of BM LT-HSCs, ST-HSCs and MPP cells in vivo (Supplementary Fig. 2B, C). However, *Phf6*-deficient HSCs had much stronger hematopoietic regeneration capacity than *Phf6 WT* HSCs in competitive bone marrow transplantation assay (Supplementary Fig. 2D). Notably, *VC Phf6* and *Phf6 WT* mice barely showed any disease symptoms or pathological phenotypes in the PB, BM and spleen (Supplementary Fig. 2E). These results suggested that PHF6 is essential for the proliferation of myeloid leukemia cells, but dispensable for normal hematopoiesis.

### Deletion of *Phf6* decreased leukemia progression in RE9a- and MA9-driven AML mouse models

**Fig. 2:**
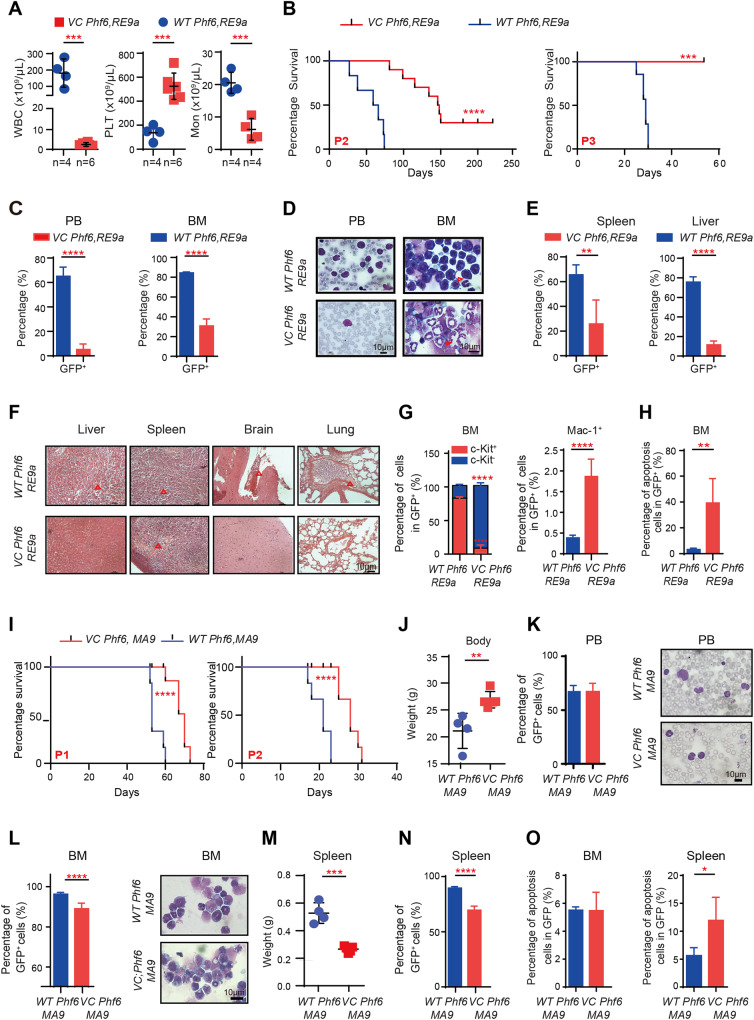
Deletion of *Phf6* delayed RE9a- and MA9-driven leukemia development. **A** WBC, PLT, and monocyte (Mon) counts in PB by routine blood tests. **B** Kaplan–Meier survival curves of *VC Phf6,RE9a* and *WT Phf6,RE9a* AML mice in passage 2 to 3 (log-rank test *p* < 0.005, n ≥ 5 per group). **C** Percentage of GFP^+^ cells in PB and BM of *VC Phf6,RE9a* and *WT Phf6,RE9a* AML mice. **D** Wright-Giemsa staining of PB cells and BM cells. **E** Percentage of GFP^+^ cells in spleen and liver of *VC Phf6,RE9a* and *WT Phf6,RE9a* AML mice. **F** HE staining of spleen, liver, lung, and brain of *VC Phf6,RE9a* and *WT Phf6,RE9a* AML mice. The red triangle indicated the leukemia cell infiltration area. **G** The percentage of GFP^+^c-Kit^+^ and Mac-1^+^ cells in BM of *VC Phf6,RE9a* and *WT Phf6,RE9a* AML mice. **H** The percentage of apoptotic AML cells in BM of *VC Phf6,RE9a* and *WT Phf6,RE9a* mice. **I** Kaplan-Meier survival curves of *VC Phf6,MA9* and *WT Phf6,MA9* AML mice in passage 1 and 2 (log-rank test *p* < 0.005, *n* ≥ 5 per group). **J** The body weight of *VC Phf6,MA9* and *WT Phf6,MA9* AML mice. **K** The percentage of GFP^+^ cells in PB and Wright-Giemsa staining of PB cells. **L** The percentage of GFP^+^ cells in bone marrow and Wright-Giemsa staining of BM cells. **M**, **N** The weight and the percentage of GFP^+^ cells of spleen in *VC Phf6,MA9* and *WT Phf6,MA9* AML mice. **O** The percentage of apoptotic AML cells in BM and spleen of *VC Phf6,MA9* and *WT Phf6,MA9* mice. Data are represented as mean ± SD.

To determine the impact of Phf6 deletion in leukemogenesis in vivo, we utilized two mouse models that expressed RUNX1-ETO9a (RE9a) or MLL-AF9 (MA9) in hematopoietic cells. We sorted Lin^-^ cells from E14.5 fetal liver cells or bone marrow of *VC Phf6* or *Phf6*^*fl/y*^ male mice to transfect with RE9a-GFP virus or MA9-GFP virus. Equal amount of GFP^+^ cells were serially transplanted into male recipients to establish two AML mouse models (Supplementary Fig. 3A). We analyzed the phenotype of two groups when one of the groups became moribund. All *Phf6*^*fl/y*^*,RE9a* (*WT Phf6,RE9a*) mice rapidly succumbed to leukemia characterized by significantly increased white cell counts and decreased PLT counts in PB as compared to *Vav1-Cre;Phf6*^*fl/y*^*,RE9a* (*VC Phf6,RE9a)* mice in passage 3 (Fig. 2A). The survival time of all *VC Phf6,RE9a* mice was significantly longer than that of *WT Phf6,RE9a* mice in passage 1 to 3 (Fig. 2B and Supplementary Fig. 3B). The median survival of *WT Phf6,RE9a* was 62.5 days and 147 days for *VC Phf6,RE9a* in passage 2. Notably, the *VC Phf6,RE9a* mice showed much milder disease symptoms than *WT Phf6,RE9a* mice, including lower counts of GFP^+^ leukemia cells in the PB and BM (Fig. 2C, D), higher body weights and lower spleen and liver weights (Supplementary Fig. 3C). The percentage of GFP^+^ leukemia cells and the degree of extramedullary infiltration in the liver, spleen, brain and lung also decreased in *VC Phf6,RE9a* mice than that of *WT Phf6,RE9a* mice (Fig. 2E, F). Additionally, the percentage of GFP^+^ c-Kit^+^ cells was significantly decreased, while the percentage of Mac-1^+^ cells was increased in the BM of *VC Phf6,RE9a* mice when compared with that of *WT Phf6,RE9a* mice (Fig. 2G), indicating that *Phf6 KO* promoted the differentiation of RE9a-induced leukemia cells. Furthermore, we found that *Phf6 KO* promoted GFP^+^ leukemia cell apoptosis and blocked the cell cycle in the phase of G0 (Fig. 2H and Supplementary Fig. 3D). These results demonstrated that *Phf6* deficiency delayed the RE9a-induced AML progression in vivo.

We further observed the role of Phf6 in MA9-induced AML progression. Consistent with RE9a-induced AML mouse model, the survival time of *Vav1-Cre;Phf6*^*fl/y*^*,MA9* (*VC Phf6,MA9*) mice was longer than that of *Phf6*^*fl/y*^*,MA9* (*WT Phf6,MA9*) mice (Fig. 2I). The median survival of *WT Phf6,MA9* was 21 days and 28 days for *VC Phf6,MA9* in passage 2. The body weight of *VC Phf6,MA9* were higher than the control mice (Fig. 2J). The percentage of GFP^+^ leukemia cells in PB was equal in *WT Phf6,MA9* and *VC Phf6,MA9* AML mice (Fig. 2K). The percentage of GFP^+^ leukemia cells was significantly decreased in BM of *VC Phf6,MA9* when compared with that of *WT Phf6,MA9* mice (Fig. 2L and Supplementary Fig. 3E). In addition, the weight and GFP^+^ leukemia cells of spleen were reduced in *VC Phf6,MA9* mice than that of *WT Phf6,MA9* mice (Fig. 2M, N). Furthermore, *Phf6* deletion led to more GFP^+^ leukemia cells apoptosis in the spleen but not in BM of *VC Phf6,MA9* (Fig. 2O). Taken together, these results further demonstrated that *Phf6* was essential for MA9-induced AMLs, indicating a potential general activating role of Phf6 in AMLs.

### Loss of *Phf6* decreased LSC counts and impaired LSC self-renewal

**Fig. 3:**
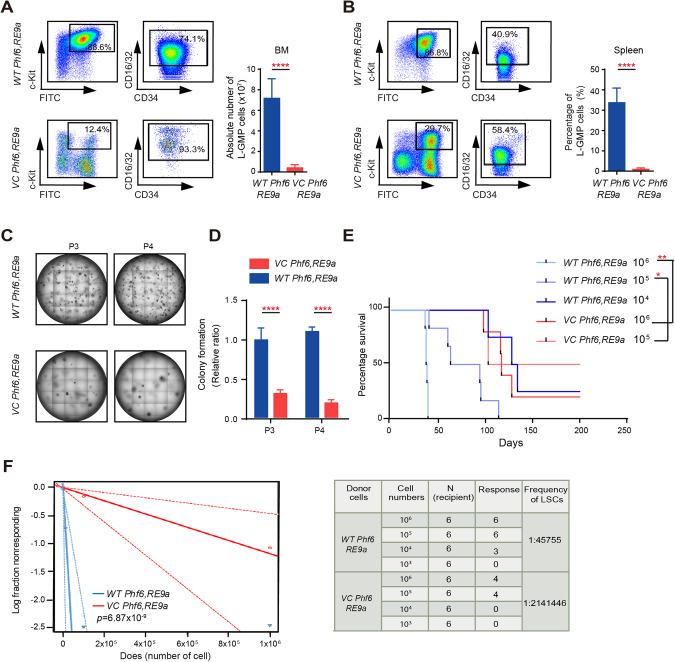
Deletion of *Phf6* decreased LSC number and impaired LSC self-renewal. **A** The absolute number of L-GMPs (GFP^+^CD34^+^CD16/32^+^) in BM of *VC Phf6,RE9a* and *WT Phf6,RE9a* AML mice. **B**. The percentage of L-GMPs in spleen of *VC Phf6,RE9a* and *WT Phf6,RE9a* AML mice. **C** Deletion of *Phf6* decreased the self-renewal capacity of RE9a-expressing fetal liver cells in serial replanting assays. **D** The number of colonies generated from 3000 RE9a-expressing cells in third and fourth plating. **E** Kaplan–Meier survival curves of *VC Phf6,RE9a* and *WT Phf6,RE9a* mice in different dilutions of leukemia cells assay (log-rank test *p* < *0.05*, *n* = 6 per group). **F** Loss of *Phf6* significantly decreases the frequency of leukemia stem cells in the limiting dilution assay. The log-fraction plot (left panel) showed the results of the limiting dilution assay by using different dilutions of leukemia cells from *VC Phf6,RE9a* and *WT Phf6,RE9a* in vivo (right panel). Data are represented as mean ± SD.

The L-GMP cells (Lin^-^c-Kit^+^sca-1^-^CD16/32^+^) were defined as LSCs in AML mouse model. To determine the consequence of *Phf6* loss on maintenance of RE9a- and MA9-transformed LSCs in vivo, we characterized the L-GMPs in two AML mouse models when the AML mice became moribund. We found the number or the percentage of LSCs were decreased in BM, spleen and liver of *VC Phf6,RE9a* mice as compared with that of *WT Phf6,RE9a* mice (Fig. 3A, B and Supplementary Fig. 4A, B). We then performed serial replanting assays to examine the effect of loss of *Phf6* in self-renewal capacity of LSCs in vitro, and found that *VC Phf6,RE9a* fetal liver cells gave rise to 60% fewer colonies than *WT Phf6,RE9a* fetal liver cells at the third and fourth round of replanting assays (Fig. 3C, D and Supplementary Fig. 4C). Consistent with RE9a-induced AML mouse model, the percentage of L-GMPs in BM and spleen of *VC Phf6,MA9* were lower than that of *WT Phf6,MA9* (Supplementary Fig. 4D, E). In the serial replanting assays, the colony number of *VC Phf6,MA9* BM cells was also decreased when compared with that of the control cells (Supplementary Fig. 4F).

To quantify the functional LSCs in vivo, we performed extreme limiting dilution transplantation assays with AML cells from *VC Phf6,RE9a* or *WT Phf6,RE9a* mice. The recipient mice were monitored for survival for 4 months post-transplantation. The recipient mice received *WT Phf6,RE9a* AML cells developed AML with a shorter latency as compared with the mice received *VC Phf6,RE9a* cells (Fig. 3E). As determined by ELDA software, the frequency of LSCs was reduced by 47-fold in recipient mice transplanted with *VC Phf6,RE9a* AML cells as compared with the control mice (Fig. 3F). In summary, these data indicated that loss of *Phf6* effectively delayed the AML progression by impairing the self-renewal capacity of LSCs.

### Lack of *Phf6* promoted AML cells apoptosis by inhibiting NF-κB signaling pathway

**Fig. 4:**
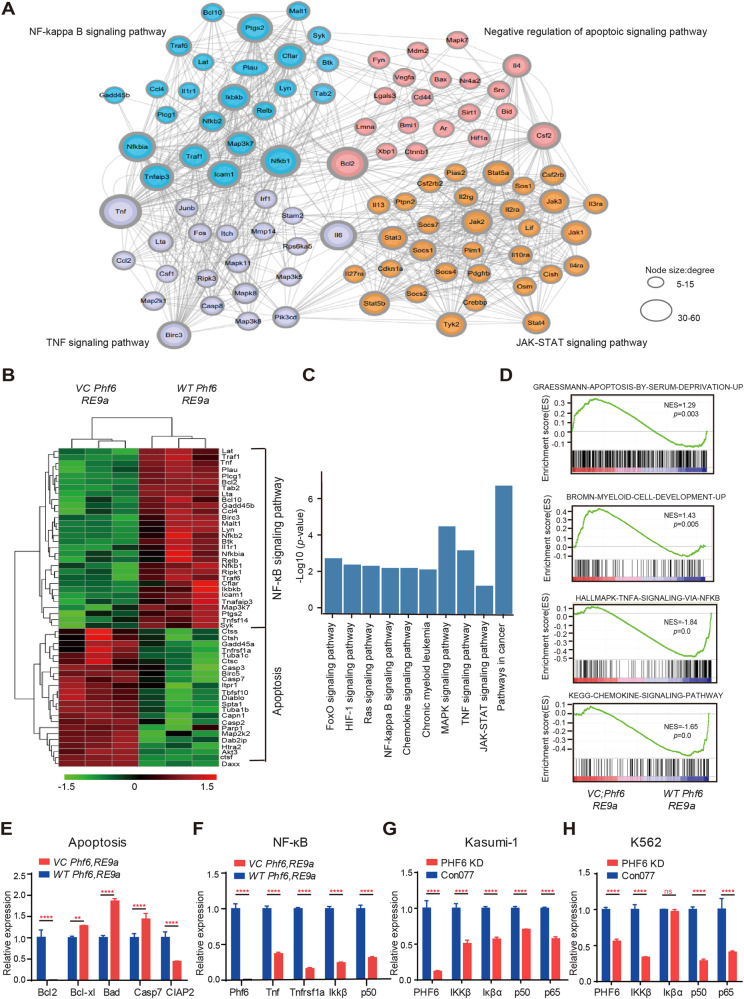
*Phf6* loss changed the transcriptional profile of apoptosis-related and NF-κB-related signaling pathways in AML cells. **A** Gene interaction analysis showing the significantly altered expression pattern in genes that took part in the NF-κB signaling pathway, TNFα signaling pathway, JAK-STAT signaling pathway and apoptotic signaling pathway in *VC Phf6,RE9a* AML cells when compared with *WT Phf6,RE9a* AML cells. **B** The heatmap of apoptosis-related and NF-κB related genes. **C** KEGG analysis of downregulated signaling pathway in *VC Phf6,RE9a* AML cells when compared with *WT Phf6,RE9a* AML cells. **D** Gene set enrichment analysis (GSEA) of *VC Phf6,RE9a* AML cells versus *WT Phf6,RE9a* AML cells. **E**, **F** Validation of the expression of apoptosis-related and NF-κB-related genes in *VC Phf6,RE9a* and *WT Phf6,RE9a* AML cells. **G**, **H** The expression of NF-κB related genes in PHF6 KD Kasumi-1 and PHF6 KD K562 cells. Data are represented as mean ± SD.

To explore the underlying molecular mechanisms of *Phf6* loss in delaying AML progression, we analyzed the transcriptional profiles of *VC Phf6,RE9a* and *WT Phf6,RE9a* AML cells via RNA-sequencing. It revealed a distinct gene expression signature in *VC Phf6,RE9a* cells (870 genes upregulated and 961 genes downregulated; *p* < 0.05, Supplementary Fig. 5A). Differentially expressed genes were significantly enriched for apoptosis, NF-κB, TNFα and JAK-STAT signaling pathway (Fig. 4A). 23 apoptosis-related genes were upregulated, and 30 NF-κB-related genes were downregulated in *Phf6* KO leukemia cells (Fig. 4B). GO and KEGG analysis showed that leukemogenesis-related pathways were downregulated, such as ERK, JAK-STAT, NF-κB, and RAS signaling pathway et al. (Fig. 4C and Supplementary Fig. 5B). Myeloid cells development and homeostasis-related pathways were upregulated in *VC Phf6,RE9a* cells (Supplementary Fig. 5C, D). GSEA showed upregulation of apoptosis and myeloid cells development-related genes, and downregulation of TNFα-NF-κB and chemokine-regulated genes in *VC Phf6,RE9a* cells (Fig. 4D). We further validated apoptosis-related genes found that anti-apoptosis gene Bcl2 and CIap2 were downregulated, while genes promoting apoptosis Bad and Casp7 were upregulated in *VC Phf6,RE9a* cells (Fig. 4E). Notably, *Phf6* loss inhibited the NF-κB signaling pathway, as validated by the decreased mRNA expression of Tnf, Ikkβ and p50 in *VC Phf6,RE9a* AML cells when compared with *WT Phf6,RE9a* AML cells (Fig. 4F). Moreover, we also confirmed that PHF6 KD by shRNA1 reduced the mRNA expression of NF-κB related genes in Kasumi-1 and K562 cells (Fig. 4G, H). Thus, it suggested that Phf6 depletion might promote AML cell apoptosis by blocking the NF-κB signaling pathway.

### Depletion of *PHF6* inhibited NF-κB activity by disrupting the PHF6-p50 transcriptional complex and blocking p50 nuclear translocation

**Fig. 5:**
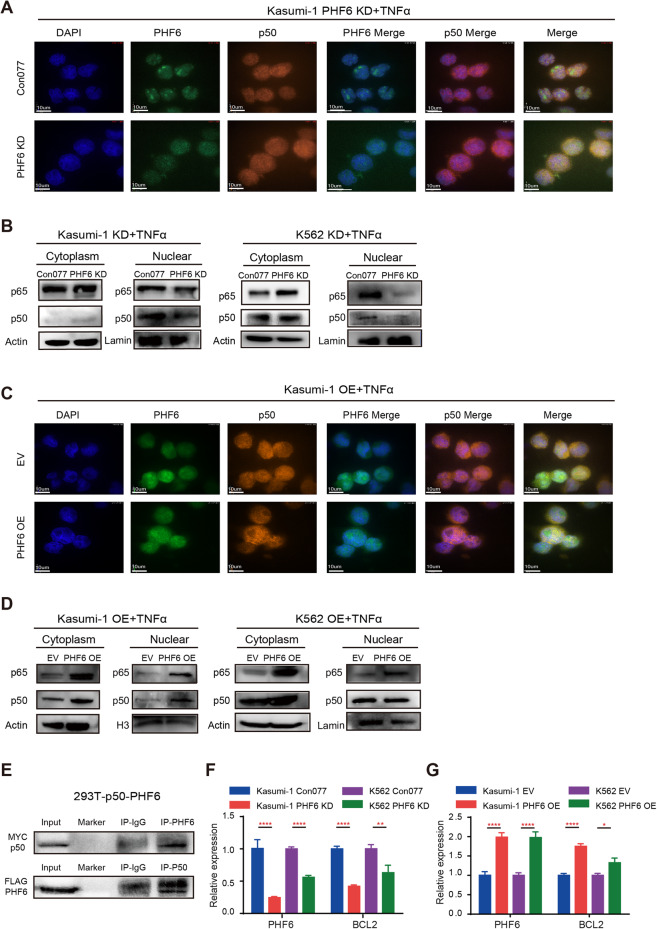
*Phf6* deletion inhibited NF-κB activity by decreasing p50 nuclear translocation. **A** Nuclear translocation of p50 (Orange) in PHF6 KD Kasumi-1 cells after TNFα stimulation by immunocytochemistry assay. Nuclei were identified using DAPI (Blue). Scale bars, 10 μm for Kasumi-1 cells. Data were representative of three independent experiments. **B** The protein expression of p50 and p65 in the nucleus and cytoplasm of PHF6 KD Kasumi-1 and PHF6 KD K562 cells in presence of TNFα. The cells were stimulated by 100 ng/ml TNFα for 2 h. **C** The co-location of PHF6 (Green) and p50 (Orange) in PHF6 OE Kasumi-1 cells after TNFα stimulation by immunocytochemistry assay. Nuclei were identified using DAPI (Blue). Scale bars, 10 μm for Kasumi-1 cells. **D** The protein expression of p50 and p65 in the nucleus and cytoplasm fractions of PHF6 OE Kasumi-1 and PHF6 OE K562 cells in presence of TNFα. **E** PHF6-FLAG and p50-MYC were overexpressed in 293 T cells (293T-p50-PHF6 cells). Upper panel, Co-IP was performed with FLAG antibody. The p50 was determined by MYC antibody in 293T-p50-PHF6 cells. Lower panel, Co-IP was performed with MYC antibody. The PHF6 was determined by FLAG antibody in 293T-p50-PHF6 cells. **F** The mRNA expression of PHF6 and BCL2 in PHF6 KD Kasumi-1 and PHF6 KD K562 cells. **G** The mRNA expression of PHF6 and BCL2 in PHF6 OE Kasumi-1 and PHF6 OE K562 cells. Data are represented as mean ± SD.

To investigate the mechanisms by which PHF6 regulated NF-κB activity, we assessed the expression of NF-κB related factors in PHF6 KD by shRNA1 myeloid leukemia cells and control cells by Western blotting analysis. We found that the expression of p-IKKβ/IKKβ, p50 and p-p65/p65 were not altered in PHF6 KD Kasumi-1 and K562 cells when compared with their respective controls in the presence or absence of TNFα (Supplementary Fig. 5E, F). The expression of p-IκBα/IκBα were slightly higher in PHF6 KD Kasumi-1 cells than the control cells, while it was similar in PHF6 KD K562 cells and control cells (Supplementary Fig. 5E, F). Since the nuclear translocation of p50 and p65 directly regulates NF-κB downstream target genes, we further assessed the nuclear translocation of p50 and p65 in PHF6 KD myeloid leukemia cells in response to TNFα treatment by immunofluorescence. We found that p50 translocated into the nucleus of Kasumi-1 and K562 control cells successfully, while it retained in the cytoplasm of PHF6 KD Kasumi-1 and K562 cells after treatment of TNFα (Fig. 5A and Supplementary Fig. 6A, B). We did not observe any difference in the nuclear translocation of p65 in PHF6 KD myeloid leukemia cells and control cells after TNFα treatment (Supplementary Fig. 6C). Notably, the overall amount of p50 was similar in PHF6 KD myeloid leukemia cells and control cells in the presence or absence of TNFα, while the nuclear p50 and p65 significantly decreased after TNFα treatment in PHF6 KD Kasumi-1 and K562 cells when compared with the control cells respectively (Fig. 5B). Furthermore, we found that PHF6 OE led to more p50 translocated from the cytoplasm to the nucleus under the stimulation of TNFα. In this process, PHF6 accompanied p50 into the nucleus and eventually co-localized in the nucleus of PHF6 OE Kasumi-1 and K562 cells (Fig. 5C and Supplementary Fig. 7A, B). Western blot assay also showed that PHF6 OE induced more p50 translocated into the nucleus in Kasumi-1 cells and more p65 translocated into the nucleus in K562 cells (Fig. 5D). These data indicated that PHF6 regulated the NF-κB activity via the nuclear translocation of p50.

To further investigate whether PHF6 directly bound with p50 and regulated the function of p50, we constructed 293T-p50-PHF6 cells, in which PHF6-FLAG (carry 3xFlag-HA tag) and p50-MYC (carry 6xHis-MYC tag) were overexpressed (Supplementary Fig. 7C). Co-immunoprecipitation experiments confirmed a physical interaction of PHF6 and p50 reciprocally (Fig. 5E). It has been reported that p50 directly bound to *BCL2* gene and promoted the mRNA transcription of BCL-2 [31]. We thus measured the mRNA expression of BCL2 in PHF6 KD cells, PHF6 OE cells and control cells. We found that BCL2 was decreased in PHF6 KD Kasumi-1 and PHF6 KD K562 cells when compared with that of control cells (Fig. 5F). Consistently, BCL2 was increased in PHF6 OE Kasumi-1 and PHF6 OE K562 cells when compared with that of control cells (Fig. 5G). These data demonstrated that PHF6 deficiency blocked NF-κB signaling pathway by disrupting the PHF6-p50 transcriptional complex and partially inhibiting the p50 nuclear translocation.

### Inhibition of NF-κB could suppress PHF6 OE-induced AML progression

**Fig. 6:**
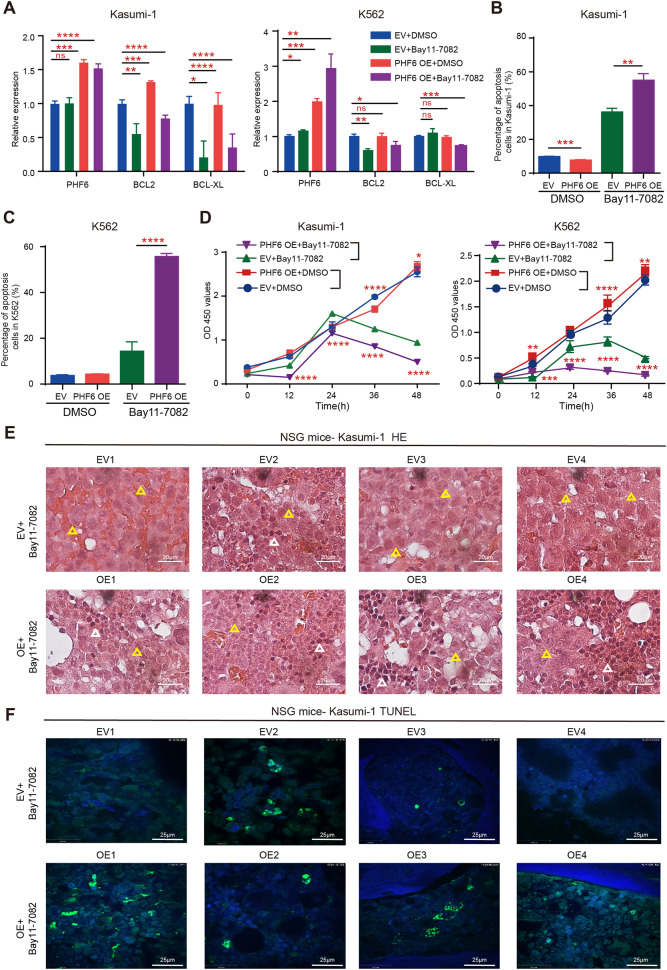
NF-κB inhibitor Bay11-7082 could rescue the over-proliferation of PHF6 OE Kasumi-1 cells in vitro and in vivo. **A** The mRNA expression of BCL2 and BCL-XL in PHF6 OE Kasumi-1, PHF6 OE K562 and control cells (EV) after treatment with Bay11-7082 (4 μM) or DMSO. **B**, **C** The percentage of apoptotic cells in PHF6 OE Kasumi-1, PHF6 OE K562 and control cells after treatment with Bay11-7082 or DMSO. **D** The proliferation of PHF6 OE Kasumi-1, PHF6 OE K562 and control cells after treatment with Bay11-7082 or DMSO. **E**, **F** Histological staining for hematoxylin and eosin (HE) (Yellow triangles represents Kasumi-1 cells, White triangles represents bone marrow cells, magnification, 100×) and TUNEL staining (green represents TUNEL, blue represents DAPI, magnification, 60×) in BM from NSG mice transplanted with PHF6 OE Kasumi-1 or control cells (EV), and treated with Bay11-7082. Data are represented as mean ± SD.

To determine whether inhibition of NF-κB could delay the over-proliferation of PHF6 OE AML cells, we treated PHF6 OE myeloid leukemia cells with BAY11-7082 (a selective NF-κB inhibitor). We found that BAY11-7082 effectively reduced the mRNA expression of BCL2 and BCL-XL in PHF6 OE Kasumi-1 and K562 cells when compared with cells treated with DMSO (Fig. 6A), indicating that BAY11-7082 inhibited the activity of NF-κB signaling pathway. The percentage of apoptotic PHF6 OE Kasumi-1 and K562 cells were much higher than the cells treated with DMSO (Fig. 6B, C). Furthermore, we found that BAY11-7082 significantly inhibited the growth of PHF6 OE Kasumi-1 and K562 cells in vitro (Fig. 6D).

Next, we assessed the anti-leukemia efficacy of the NF-κB inhibitor on PHF6 OE AML cells in vivo. We transplanted PHF6 OE Kasumi-1 or control cells into 200cGY irradiated NSG mice and treated these mice with BAY11-7082 after a month. Tumor burden was quantified using Hematoxylin and Eosin (HE) staining of BM. Upon treatment with BAY11-7082 of three times in 10 days, the mice engrafted with PHF6 OE Kasumi-1 cells showed slight infiltration of human AML cells (Yellow arrows) in BM than that of mice engrafted with control cells (Fig. 6E and Supplementary Fig. 7D), indicating that BAY11-7082 could partially rescue the over-proliferation of PHF6 OE Kasumi-1 cells in vivo. In addition, we found that apoptotic cells were increased in BM of mice engrafted with PHF6 OE Kasumi-1 cells than that of mice engrafted with control cells by the TUNEL (TdT-mediated dUTP nick-end labeling) staining (Fig. 6F). These results demonstrated that therapy with NF-κB inhibitor could reduce the progression of PHF6 OE-induced AML by promoting leukemia cells apoptosis in vivo.

## Discussion

AML is a major hematopoietic malignancy characterized by the uncontrolled expansion of the immature myeloid cells [1]. Myeloid leukemia exhibited a dysregulated developmental programing initiated by both genetic and epigenetic alterations. Understanding the underlying mechanisms is prerequisite for the successful development of leukemia cells-targeted therapeutic agents. Here in this study, we showed that PHF6 played a pro-oncogenic role in myeloid leukemia development induced by RUNX1-ETO9a and MLL-AF9 in mice, while PHF6 is not essential for normal hematopoiesis, suggesting it might be an attractive drug target.

The PHF6 is a conserved epigenetic regulator which plays important roles in hematopoiesis and leukemogenesis [16, 32, 33]. Wendorff et al. showed that loss of *Phf6* led to the expansion of HSCs in mice, but observed no differences in the number of myeloid progenitors, lymphoid progenitors or B-cell precursors [23]. Although *PHF6* mutations often occurred in leukemia patients [18, 24, 27], *PHF6* deficiency alone was not sufficient to induce leukemic transformation [34]. It has been reported that Phf6 loss could significantly accelerate T-ALL development driven by co-mutation with JAK3^M511I^ or with aberrant expression of TLX3, suggesting PHF6 as a tumor suppressor in T-ALL [21, 23]. Interestingly, PHF6 mutation rates are significantly lower in B-ALL and AML patients when compared with that of T-ALL patients [24, 35], and in contrast to its role in T-ALL, PHF6 appears to play a pro-oncogenic role in B-ALL and AML. Depletion of Phf6 significantly decreased the growth of leukemia cells in Em-Myc B-ALL mouse model and prolonged the survival of BCR-ABL induced B-ALL mouse model in vivo [35, 36]. Mousa et al. determined that PHF6 was upregulated in AML patients [28, 37]. In consistent with their findings, we found that high level of PHF6 predicted an unfavorable prognosis for AML patients, and promoted the growth of myeloid leukemia cells (Fig. 1 and Supplementary Fig. 1). These independent studies indicated that PHF6 may have lineage-specific roles in hematologic malignancies, and possibly via different signaling pathways in different malignancies.

**Fig. 7:**
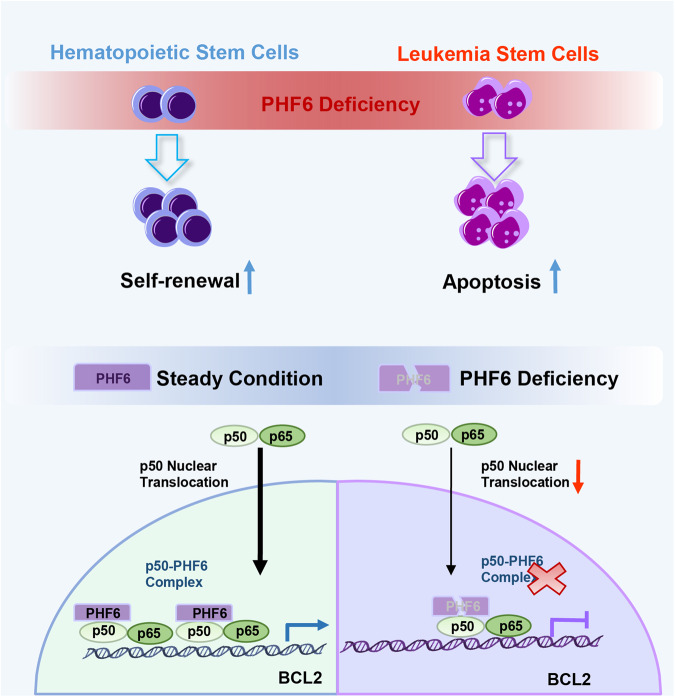
Schematic diagram of PHF6-p50 co-transcriptional complex to regulate NF-κB signaling pathways. In steady condition, PHF6 binds to p50 and forms a PHF6-p50 co-transcriptional complex to regulate p50 translocation into nucleus, and to promote transcription of BCL2. Under PHF6 deficient condition, the complex of PHF6-p50 is disrupted, p50 nuclear translocation decreased, the expression of BCL2 reduced, leukemia cells apoptosis increased and the AML progression delayed.

In our study, we found that overexpression of PHF6 in Kasumi-1 cells with RUNX1-ETO mutation and K562 cells with a BCR-ABL1 mutation accelerated the growth of cells. On the other hand, deletion of Phf6 delayed the progression of AML in both RE9a and MA9 mouse models, as knock-down of PHF6 impaired the growth of Kasumi-1 and THP1 cells. It suggested that high PHF6 expression could play a general supporting role in all subtypes of myeloid leukemia. We speculated that PHF6 might contribute to leukemogenesis of AML through additional signaling pathways, not limited to RE9a- or MA9-pathways we investigated in this study. Notably, our mRNA profiling analysis demonstrated that Phf6 loss activated the apoptosis-related signaling pathways and inhibited NF-κB signaling pathways in AML cells (Fig. 4). It has been reported that NF-κB is constitutively activated in most acute myeloid leukemia that contribute to the resistance to leukemia cell apoptosis [38–40]. Consistent with this, we found that depletion of PHF6 increased the apoptosis of AML cells through inhibiting the NF-κB signaling pathway. The activation of canonical NF-κB pathway leads the degradation of IκBα by sequential phosphorylation, ubiquitination, and proteasome-mediated proteolysis to release the p50-p65 heterodimer, which translocates to the nucleus and binds to specific consensus sequences within the promoter of NF-κB target genes (e.g., BCL2 or BCL-XL) [41]. In current study, we showed that PHF6 could directly bind with p50 and promote the nuclear translocation of p50, thus increase the expression of BCL2 and inhibit the apoptosis of AML cells (Fig. 7), highlighting a new functional aspect of PHF6 in AML cells.

AML was maintained by a pool of LSCs endowed with self-renewal capacity and other stem-like properties that are important for the resistance of leukemia treatment [42]. Here, we found that deletion of Phf6 impaired the proliferation and self-renewal of LSCs in AML, thereby delaying leukemogenesis. It was reported that NF-κB signaling was more active in LSCs than in normal HSCs [40, 43]. Our studies showed that high level of PHF6 could aberrantly activate NF-κB signaling, which exerted anti-apoptotic and proliferation-promoting effects via BCL-2 and BCL-XL (Fig. 6). The proliferative advantage conferred by PHF6 and NF-κB might contribute to the clonal evolution of pre-leukemia cells that lead to leukemia transformation. In addition, PHF6 over-expressing myeloid leukemia cells were more sensitive to NF-κB inhibitor (BAY11-7082) than the other cells, indicating that NF-κB signaling pathways might be a new suppressive target for treatment of myeloid leukemia patients with PHF6 high expression.

In summary, our study identified an unexpected leukemogenic role of PHF6 in AMLs. We demonstrated that PHF6 is required for myeloid leukemia cell survival and LSC self-renewal via NF-κB signaling pathway. AML cells were exquisitely sensitive to *PHF6* genetic loss, while deficiency of PHF6 has little impact on normal hematopoiesis. Our study suggested that inhibiting PHF6 through blocking NF-κB signaling pathway would be a potential targeted therapeutic strategy for AML patients.

## Supplementary information

Supplementary materials

Supplementary figure1

Supplementary figure2

Supplementary figure3

Supplementary figure4

Supplementary figure5

Supplementary figure6

Supplementary figure7

Supplementary excel-patients

## Data Availability

The RNA-sequencing datasets generated during this study are available at the Gene Expression Omnibus database under accession number GSE205133.
