# Supplementary material for: PHF6 maintains acute myeloid leukemia via regulating NF-κB signaling pathway
Source: Leukemia. 2023 Jul 1;37(8):1626–37. doi: 10.1038/s41375-023-01953-6 (PMC10400421; doi:10.1038/s41375-023-01953-6)
Supplement: Supplementary file 1 — Supplementary materials [file 41375_2023_1953_MOESM1_ESM.docx]

Supplementary Data for Shuaibing Hou *et al.*: ***PHF6* maintains acute myeloid leukemia via regulating NF-κB signaling pathway** (Including Supplementary methods and 7 Supplementary Figures)

**Supplementary Methods**

**Patient samples**

Six Primary AML patients and three normal human BM samples were obtained from State Key Laboratory of Experimental Hematology (SKLEH), Institute of Hematology and Blood Disease Hospital and Tianjin Second Hospital, Tianjin Medical University. The specific patient information was in supplementary excel data sheet 1. The informed consents of patient sample usage were obtained, and Experiments were approved by the Ethic Committee of the Hospital (KT2020052-EC-1) .

**Cell lines and culture**

Kasumi-1 cells, K562 cells, THP1 cells and HEK293T cells were obtained from cell bank of SKLEH (purchased from ATCC and maintained by the cell bank). K562 and THP1 cells were grown in 1640 media (Gibco, C11875500BT, CA, USA) with 10% fetal bovine serum (Biological Industries, 04-001-1ACS, Israel or Gibco, 10091148). HEK293T were grown in DMEM (Gibco, C11965500BT, CA, USA) media with 10% FBS. The t(8;21) positive AML cell lines Kasumi-1 were grown in RPMI 1640 media with 15% FBS (Gibco, 10091148, CA, USA). All cells were grown supplemented with 1% penicillin-streptomycin at 37℃ in a humidified 5% CO_2_ incubator. All cell lines were identified by STR and had not cross-contamination. All cell lines used the following shRNAs to knock down PHF6.

shRNA1: CCGGGCTGAGTTTGACATGTTGATACTCGAGTATCAACATGTCAAACTCAGCTTTTTG.

shRNA2: CCGGCAGAATTTGGAGACTTTGATACTCGAGTATCAAAGTCTCCAAATTCTGTTTTT.

**CD34^+^ cord blood cells purification**

Human cord blood samples were obtained from the company of Vcanbio. Briefly, white blood cells were separated from other types of blood cells by centrifugation. After the washing, CD34^+^ cells were magnetically labeled with CD34^+^ MicroBeads and then separated by MACS column. Human CD34^+^ MicroBead kit was purchased from Miltenyi Biotec Company (130-046-703, Cologne, Germany). Purified CD34^+^ cells were then cultured for 24 hours in StemSpan SFEM II (StemCell Technologies, 09655, Vancouver, Canada) with cytokine mix (FLT3 ligand 100 ng/mL; SCF 100 ng/mL; TPO 50 ng/mL; IL-3 20 ng/mL, all from Peprotech, New Jersey, USA) and 1 mM L-Glutamine.

**Proliferation and apoptosis assays**

Cell proliferation and viability were determined by cell counting and CCK-8 (Dojindo, ck-04, Kumamoto, Japan). 2000 Kasumi-1 cells, 3000 THP1 cells or 1500 K562 cells were seeded into each well of 96-well plates and cultured in 100 μl 1640 with different concentrations of FBS. Before detection, 10 μl/well cell proliferation reagent CCK-8 were added, and samples were incubated for additional 4 h at 37°C and 5% CO_2_. Cell growth curves were plotted using the normalized values of OD450, and each point represents the mean of 8 independent samples. For apoptosis, 5x10^5^ Kasumi-1 cells from early infection and starvation treatment 24h of K562 cells were washed with cold PBS and 1×Annexin V binding buffer (BD, 556454, New Jersey ,USA). Cells were vortexed and incubated for 15 min at RT in the dark with Annexin V and 7-AAD (BD). After addition of 300 μl of 1×Annexin V Binding Buffer, cells were analyzed on a BD LSRII™ (BD Biosciences, New Jersey, USA) flow cytometry analyzer.

**Colony formation and serial replanting assay**

For THP1 and Kasumi-1 cells, 2000 cells were plated on MethoCult™ H4435 Classic media (StemCell Technologies, Vancouver ,Canada) and counted in the 7 days. Human CD34^+^ cord blood cells were sorted and 1000 cells were plated on MethoCult™ H4435 media. After fourteen days, the colony number were counted. The RE9a transduced E14.5 fetal liver GFP^+^ cells from *VC Phf6,RE9a* and *WT Phf*6*,RE9a* mice were sorted and 5000 cells were plated on methylcellulose media (MethoCult™ M3231, StemCell Technologies, Vancouver ,Canada). Seven days after plating the cells, clonogenic progenitors were counted and 3000 cells were replated weekly for 4 times. For MLL-AF9 mouse model, 500 GFP^+^ cells from bone marrow of *WT Phf6,MA9* and *VC Phf6,MA9* AML mice were plated on methylcellulose media (MethoCult™ M3231) and replated weekly for 2 times. All images were taken by high-content analysis system (Operetta CLS, Perkinelmer, UK).

**Limiting dilution transplantation**

Limiting dilution experiments consisted of cohorts of mice that had received 10^6^, 10^5^, 10^4^, or 10^3^ GFP^+^ leukemia cells from *VC Phf6,RE9a* and *WT Phf6,RE9a* mice of passage 2. The mice were monitored for 4 months. We analyzed serial limited dilution leukemia stem cell data using the ELDA online resource software.

**RNA-Sequencing**

One μg RNA per sample was used for RNA sample preparations from *VC Phf6-RE9a* and *WT Phf6-RE9a* mouse cells. Transcriptome sequencing was performed on Illumina NovaSeq 6000 platform (Illumina, CA, USA) to a total target depth of 10 million 150 bp paired end reads. Differential expression analysis was performed by DESeq2 R software package.

**Quantitative real-time PCR**

RNA was extracted using the RNeasy Mini Kit (Tsingke, TSP413, China) according to the manufacturer’s protocol. cDNA synthesis was performed using a cDNA reverse transcription kit (TransGen Biotech, AT341-02, Beijing, China) according to the manufacturer’s protocol. Quantitative PCR assays were performed in 96-well Micro Amp Fast Optical Reaction Plates (Applied Biosystems, 4344904, CA, USA) using SYBR Green Mix (Roche, 4913914001, Switzerland). The signal was detected using the Step-One Plus Real-Time PCR System (QuantStudio5, Thermo, MA, USA). 18s was used as an endogenous control for gene expression assays.

**Nuclear and cytosolic fractionation and Western Blotting**

Nuclear fraction or cytoplasmic fraction were prepared using a nuclear extract kit (Beyotime biotechnology, P0027, Shanghai, China) according to the manufacturer’s instructions protocol. 5×10^6^-1×10^7^ cells were replaced with starvation medium for 24 hours and stimulated 100 ng/ml TNF-α for 2 h before cell lysis. Protein samples were subjected to SDS-PAGE. Nitrocellulose blots were incubated at room temperature for 1 hour by blocking buffer (TBST with 5% BSA or milk), followed by incubation with the indicated antibodies at 4°C overnight. After three 15-min washes with TBST, the blots were incubated with horseradish peroxidase-conjugated secondary antibody (Jackson immunoresearch, 711-035-152, Pennsylvania, USA). Immunoreactive bands were visualized using enhanced chemiluminescence substrate (Bio-Rad, CA, USA). Antibodies were used as follows: anti-AML1, anti-p-IKKβ, anti-IKKβ, anti-IKβα, anti-p-p65 (RRID:AB_331284), anti-p65, anti-NF-κB1 p105/p50 ( RRID:AB_2665516), anti-H3, anti-β-actin, anti-Lamin and anti-GAPDH (all from Cell Signaling Technology, MA, USA), Rabbit antibody against PHF6 (Novus, Colorado, USA, NB100-79861, RRID:AB_2165371).

**Co-Immunoprecipitation**

1×10^7^-2×10^7^ cells were washed with ice-cold PBS and lysed in buffer with protease inhibitor for 15min (1% NP40, 10% glycerol, 135mM NaCl, 20mM 1M Tris-HCl, pH=8.0). Insolvable debris was removed by centrifugation. Following preclearing of Protein A/G (Thermo Scientific, 88803, MA, USA) and antibodies, an equal amount of cell lysate was subjected to the incubation with antibodies overnight at 4°C. After three washes by buffer, bound proteins were eluted with protein loading buffer and boiled at 100℃. Antibodies used in the Co-IP assay were as follows: anti-PHF6 antibody (Abcam, ab173304, Cambridge , UK), anti-MYC antibody (Cell Signaling Technology , MA, USA , 2276, RRID:AB_331783), anti-FLAG antibody (Sigma-Aldrich, Missouri,USA, F1804, RRID:AB_262044), anti-p50 antibody (Santa Cruz Biotechnology , Texas, USA, sc-8414, RRID:AB_628015).

**TUNEL staining assay**

TdT-mediated dUTP nick-end labeling (TUNEL) staining kit was used to perform experiments on paraffin-embedded sections (Beyotime, Shanghai , China). 50 µL of TUNEL reaction mixture was added to each slide after treatment with protease K and 0.1% Triton X-100. The samples were incubated in the dark in a humidified chamber for 1 -2h at 37°C and observed on microscope (UltraVIEW VOX, Perkinelmer, UK).

**Flow cytometry analysis**

Single-cell suspensions were prepared from peripheral blood, bone marrow, spleen or liver. A 10 μl peripheral blood (PB) sample was obtained from the tail vein and diluted with PBE (PBS with 2% fetal bovine serum and 2 mM EDTA). Before staining, ammonium chloride-potassium bicarbonate was used to lyse the red blood cells (RBCs). Bone marrow (BM) cells were flushed out from tibias, femurs and ilia with PBE. All antibodies were purchased from either eBioscience or Invitrogen (USA). The detail information for the antibodies was listed in the Supplementary Table 1. The samples were analyzed with a BD LSRII™ flow cytometry analyzer. More than 10000 cells were collected and the results were analyzed with FlowJo software (RRID:SCR_008520).

**Statistical analysis**

All experiments were replicated three to four times with the random numbers more than three and tests were randomized according to the design of assay. For the animal studies, all mice are randomly assigned according to the number required by phenotype and the number of mice in each experiment was chosen to provide 90% statistical power with a 5% error level. Animal experiments were not blinded and it was not necessary to be as they were less subjective. GraphPad Prism 6.0 (RRID:SCR_002798) was used for statistical analyses. Each experiment was compared as two groups. No data were excluded from the analyses.The data were presented as the mean ± standard deviation (SD). The unpaired two-tailed Student’s t test and two-away ANOVA were used to compute the *p* values. *P < 0.05* was considered significant. Kaplan-Meier curves were used to represent survival, where significance was calculated with the log rank test. Blinding was not performed because the measurements were quantitative（e.g cell numbers and percentage).

······

**Supplementary Figure legends**

**Supplementary Figure 1. PHF6 involved in AML progression .**

A. The association of PHF6 expression level with survival rate in AML patients with survival time of patients with more than one year (n=67, *p=0.0329*). B-C. The expression of PHF6 in BM MNCs of normal people and AML patients. D. Over-expression of PHF6 in Kasumi-1 and K562 cells. E. The proliferation of PHF6 OE cells in Kasumi-1 and K562 cells. F. The percentage of apoptosis cells in PHF6 OE Kasumi-1 and PHF6 OE K562 cells. G. Knock-down of PHF6 in K562 cells. H. The proliferation of PHF6 KD (shRNA1) K562 cells. I. The percentage of apoptosis cells in PHF6 KD K562 cells. J-K. The colony number of PHF6 KD Kasumi-1 and THP1 cells.

**Supplementary Figure 2. PHF6 had little impact on normal hematopoiesis.**

A. The colony number and immunophenotyping of CD34^+^ cells. B-C. The percentage of LT-HSC, ST-HSC and MPP in BM of *Vav1-Cre;Phf6^fl/y)^* (*VC Phf6*) and *Phf6^fl/y^* (*Phf6 WT)* mice. D. The percentage of CD45.2^+^ cells in *VC Phf6* and *Phf6 WT* mice. E. Wright-Giemsa staining of peripheral blood cells and BM cells and hematoxylin and eosin (HE) staining of bone, spleen in *VC Phf6* and *Phf6 WT* mice. Data are represented as mean ± SD.

**Supplementary Figure 3. *Phf6-*deficiency delayed the AML progression.**

A. The scheme of constructing RE9a and MA9 AML mouse models. B. Kaplan–Meier survival curves of *VC Phf6,RE9a* and *WT Phf6,RE9a* AML mice in passage 1 (log-rank test *p < 0.005*, n ≥ 5 per group). C. The weight of body, spleen and liver in *VC Phf6,RE9a* and *WT Phf6,RE9a* AML mice. D. The percentage of GFP^+^ BM cells at G0, G1/S, and G2/M phase in *VC Phf6, RE9a* and *WT Phf6, RE9a* AML mice. E. The flow chart of GFP^+^ and Mac-1^+^ cells in *VC Phf6,MA9* and *WT Phf6,MA9* mice. Data are represented as mean ± SD.

**Supplementary Figure 4.** **Loss of *Phf6* reduced LSC number and impaired LSC self-renewal.**

A-B. The percentage of L-GMP cells of BM and liver in *VC Phf6,RE9a* and *WT Phf6,RE9a* AML mice. C. Colony numbers in passage 1 to 4 of Lin^-^ cells from *VC Phf6* and *WT* *Phf6* E14.5 fatal livers transfected with RE9a-virus. D-E. The percentage of L-GMP cells of BM and spleen in *VC Phf6,MA9* and *WT Phf6,MA9* AML mice. F. Colony numbers of BM Lin^-^ cells from *VC Phf6,MA9* and *WT Phf6,MA9* in passage 3. Data are represented as mean ± SD.

**Supplementary Figure 5.** ***Phf6* deficiency led to inactive transcriptional profiling during AML progression.**

A. Volcano map of upregulated and downregulated genes in *VC Phf6,RE9a* and *WT Phf6,RE9a* leukemia cells. B. GO analysis about relating downregulated genes of *VC Phf6,RE9a* leukemia cells in comparison with *WT Phf6,RE9a* leukemia cells. C-D. KEGG and GO analysis about relating upregulated genes in *VC Phf6,RE9a* when compared with *WT Phf6,RE9a* leukemia cells. E-F. Protein level of NF-κB related genes in PHF6 KD Kasumi-1, PHF6 KD K562 cells and control cells in the presence or absence of 100 ng/ml TNFα.

**Supplementary Figure 6.** **PHF6 deficiency regulated the translocation of p50 but had little effect on p65 nuclear translocation.**

A. The intracellular translocation of p50 (Orange) was observed by immunofluorescence in PHF6 KD Kasumi-1 and control cells without TNFα stimulation. B. The intracellular translocation of p50 (Orange) was observed by immunofluorescence in PHF6 KD K562 and control cells in presence or absence of TNFα. C. The intracellular translocation of p65 was analyzed by immunofluorescence in PHF6 KD Kasumi-1, PHF6 KD K562 cells and control cells in the presence of TNFα. Nuclei were identified using DAPI (Blue). Scale bars, 10μm for cells. Scale bars, 10μm or 15μm for cells.

**Supplementary Figure 7. PHF6 overexpression promoted the translocation of p50 and inhibition of NF-κB signaling by Bay11-7082 showed anti-leukemia effect *in vivo*.**

A. The intracellular translocation of p50 (Orange) and PHF6 (Green) were observed by immunofluorescence in PHF6 OE Kasumi-1 and control cells without TNFα stimulation. B. The intracellular translocation of p50 (Orange) and PHF6 (Green) were observed by immunofluorescence in PHF6 OE K562 and control cells in presence or absence of TNFα. Nuclei were identified using DAPI (Blue). Scale bars, 10μm for cells. C. The exogenous protein levels of FLAG-PHF6 and MYC-p50 in 293T cells. D. Histological staining for hematoxylin and eosin (HE) (magnification, 20×) in BM from mice transplanted with PHF6 OE Kasumi-1 or control cells (EV), and treated with Bay11-7082 (Yellow triangles represents Kasumi-1 cells, White triangles represents bone marrow cells).
