# Supplementary figures and images for: PHF6 maintains acute myeloid leukemia via regulating NF-κB signaling pathway
Source: Leukemia. 2023 Jul 1;37(8):1626–37. doi: 10.1038/s41375-023-01953-6 (PMC10400421; doi:10.1038/s41375-023-01953-6)

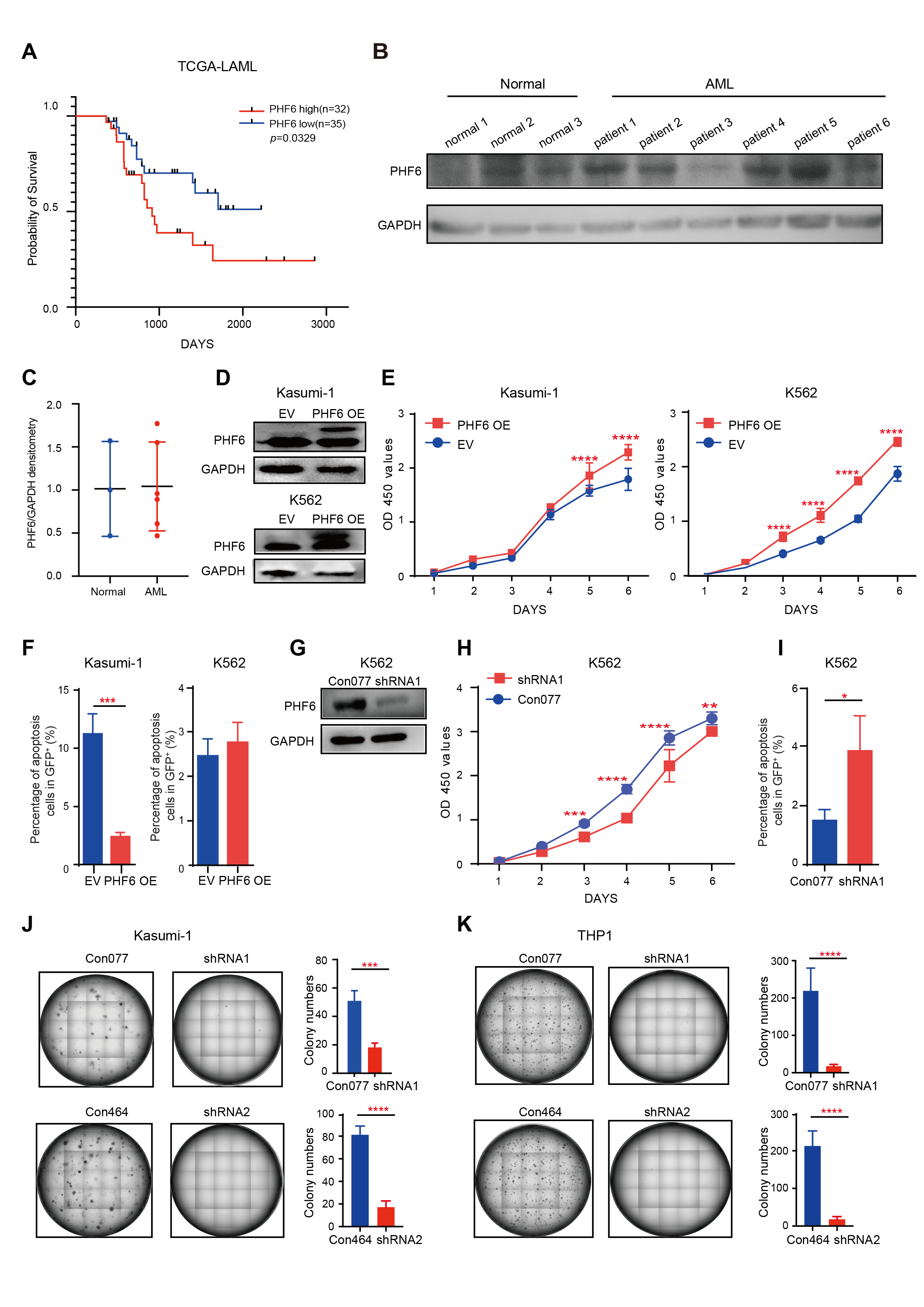

Supplement: Supplementary file 2 — Supplementary figure1 [file 41375_2023_1953_MOESM2_ESM.tif]

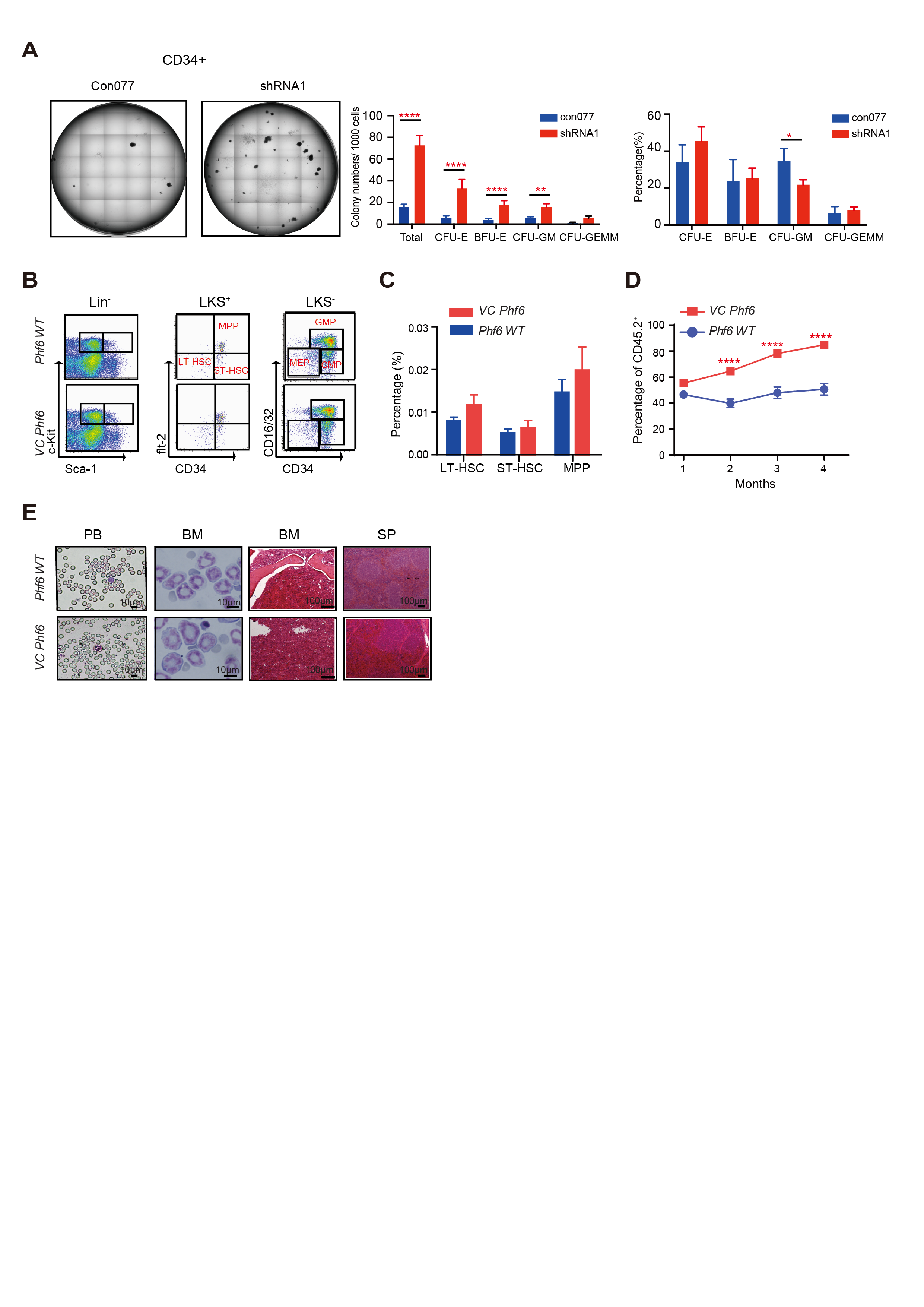

Supplement: Supplementary file 3 — Supplementary figure2 [file 41375_2023_1953_MOESM3_ESM.tif]

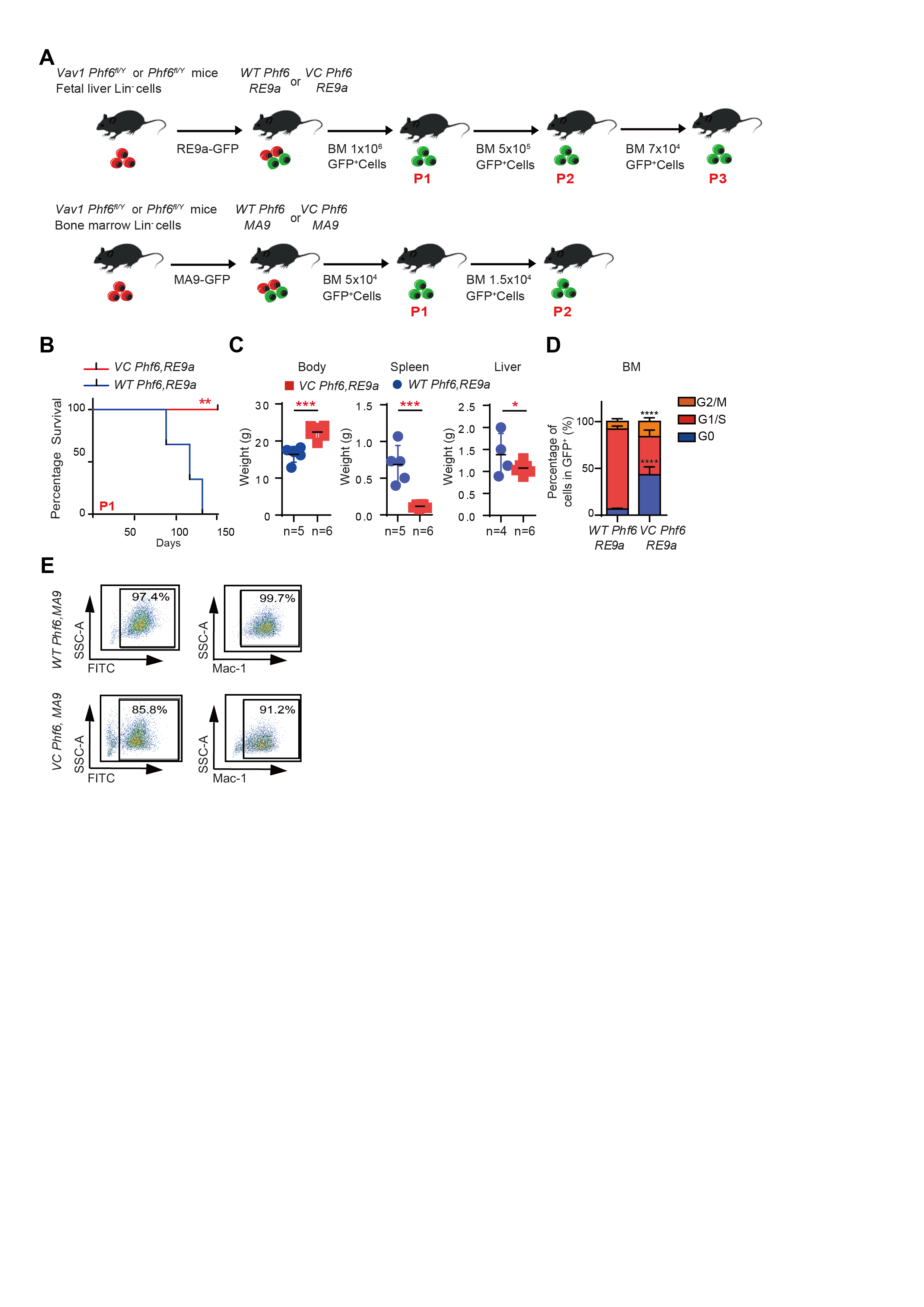

Supplement: Supplementary file 4 — Supplementary figure3 [file 41375_2023_1953_MOESM4_ESM.tif]

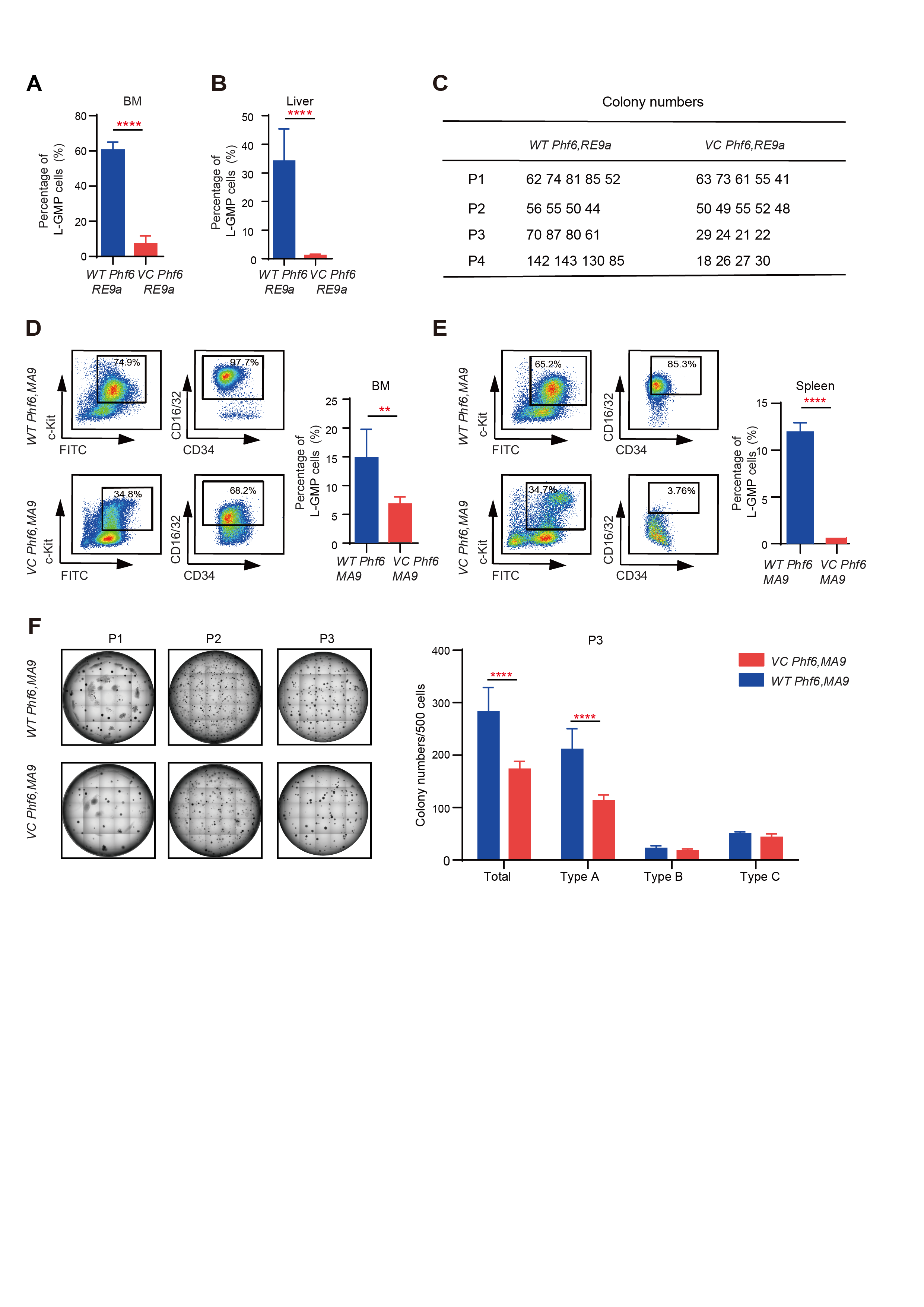

Supplement: Supplementary file 5 — Supplementary figure4 [file 41375_2023_1953_MOESM5_ESM.tif]

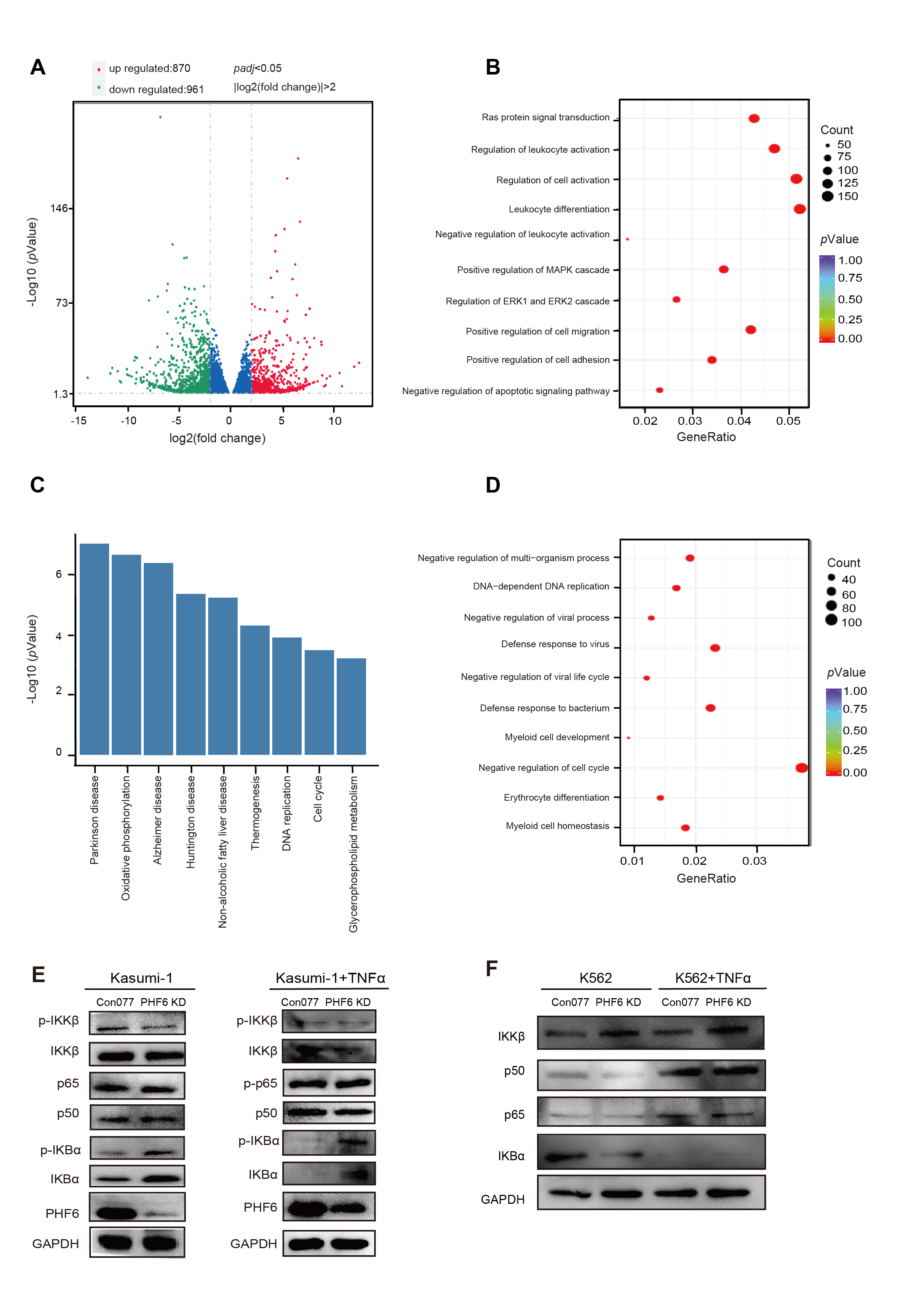

Supplement: Supplementary file 6 — Supplementary figure5 [file 41375_2023_1953_MOESM6_ESM.tif]

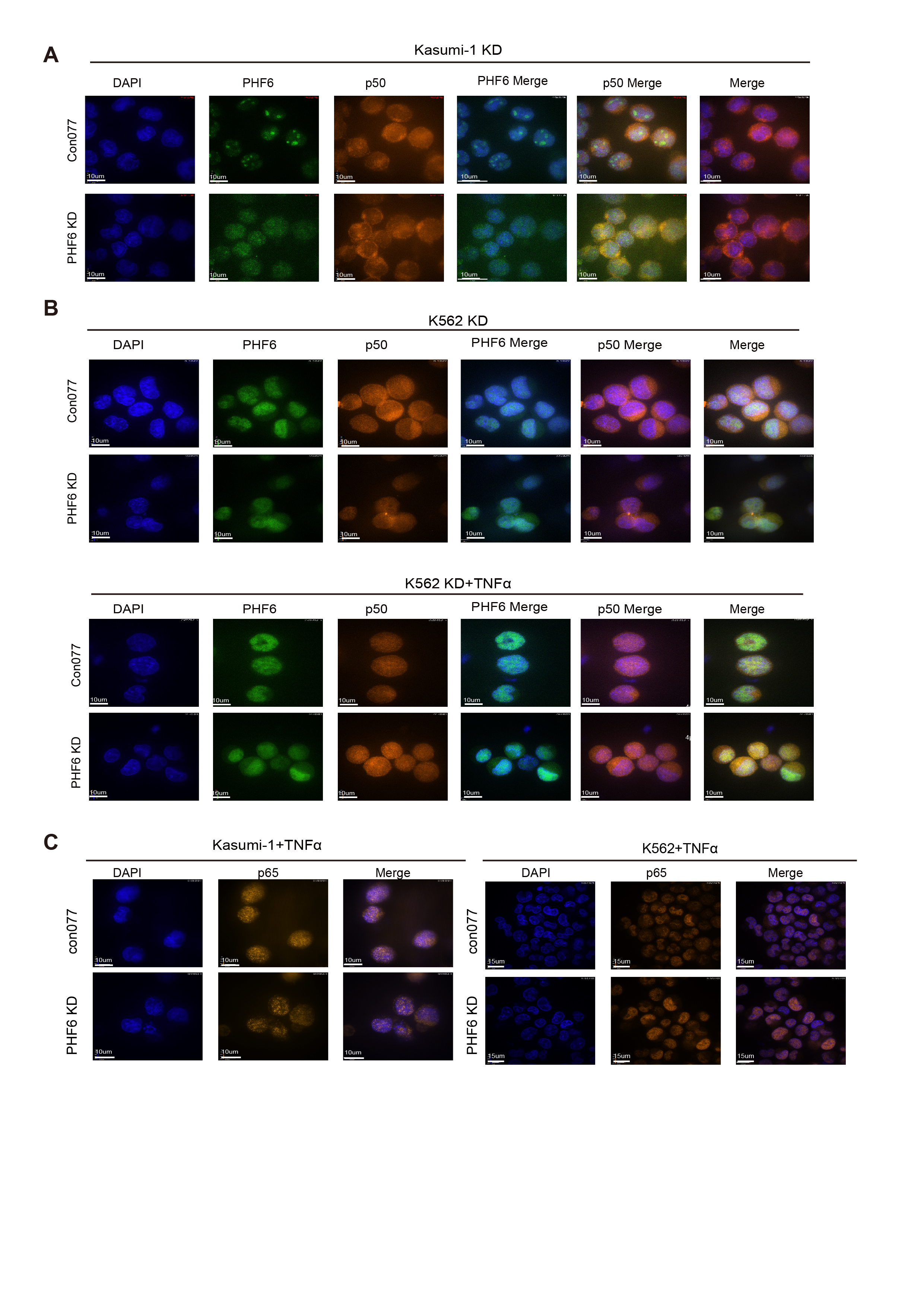

Supplement: Supplementary file 7 — Supplementary figure6 [file 41375_2023_1953_MOESM7_ESM.tif]

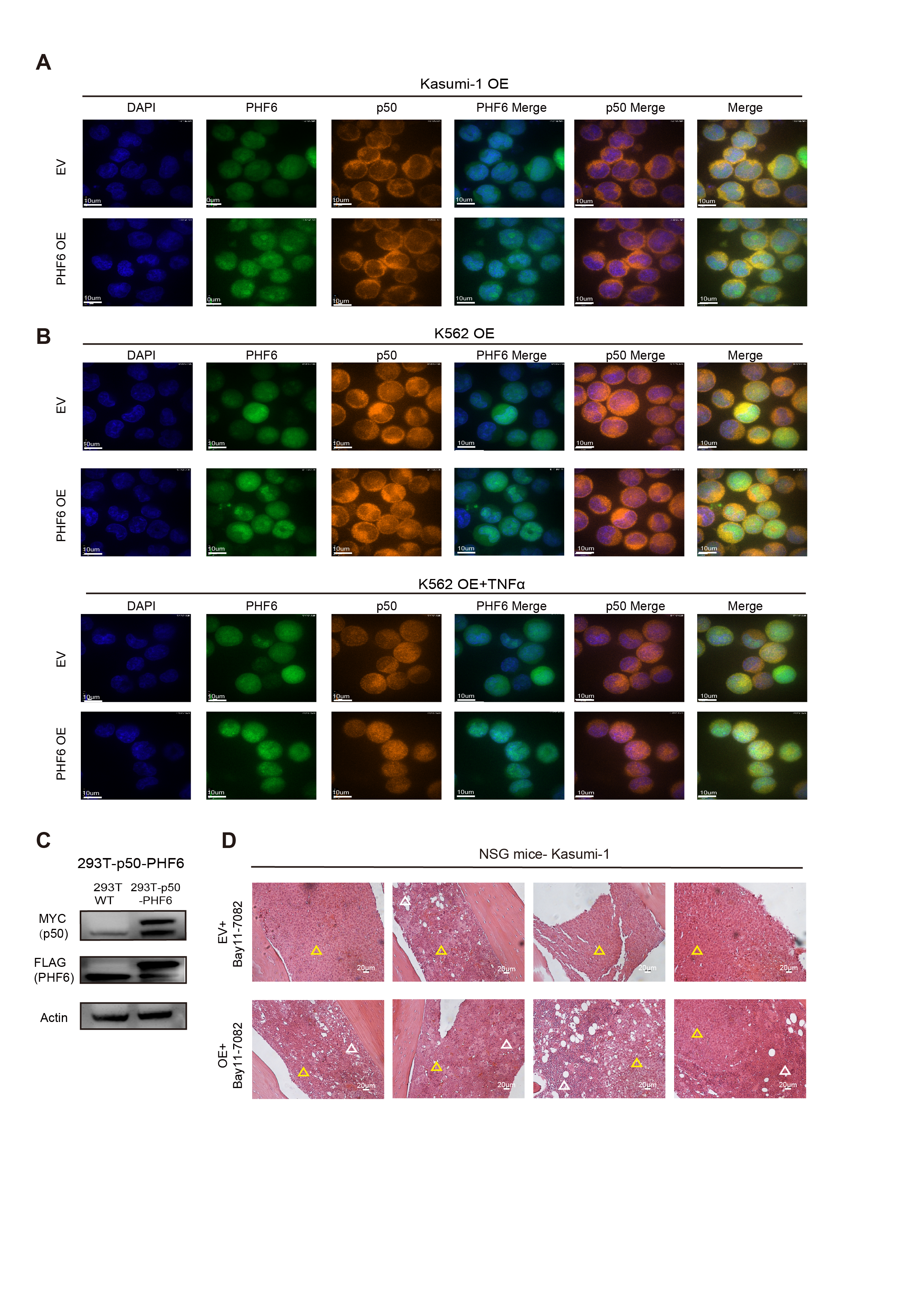

Supplement: Supplementary file 8 — Supplementary figure7 [file 41375_2023_1953_MOESM8_ESM.tif]
